# Supplementary material for: Inferring Mycobacterium bovis transmission between cattle and badgers using isolates from the Randomised Badger Culling Trial
Source: PLoS Pathog. 2021 Nov 29;17(11):e1010075. doi: 10.1371/journal.ppat.1010075 (PMC8659364; doi:10.1371/journal.ppat.1010075)
Supplement: S1 Data — Table A. Lengths of SNP alignments generated in this study. Table B. Model performance based on Maximum Likelihood Estimates (MLE) and Bayes Factors for all transmission clusters. Table C. Effective Sample Size (ESS) for TransPhylo parameters. Table D: Substitution rates for each transmission cluster. Fig A. Root to tip distances plotted against sampling dates for all isolates in each transmission cluster. Fig B. Date randomization (DTR) analysis in BEAST for each transmission cluster. Estimated substitution rates (mean and highest posterior density) shown in red for the observed dataset and black for the randomized datasets. Fig C. Pairwise distance histograms for all samples, coloured by between/within transmission cluster and separated by host pair. Fig D. Proportion of sampled and estimated unsampled cases for each transmission cluster. Sampled and unsampled cases are shown in red and blue respectively. (DOCX) [file ppat.1010075.s004.docx]

**Text. Effect of including clusters with weak temporal signal in the overall analysis**

On the basis of the DTR analysis performed on all twelve transmission clusters, two clusters, cluster 2 and 4, were shown to have weak temporal signal (the median substitution rates of one or more of the randomised datasets overlapped that of the real dataset). To assess the effect of including these clusters in the overall results, the analyses were repeated with Clusters 2 and 4 removed so a comparison between those clusters with strong or moderate temporal signal could be made with all clusters including those with weak temporal signal. The two sets of results for various analyses conducted in this study are shown below:

| Result | All clusters | Clusters 2 and 4 removed |
| --- | --- | --- |
| Substitution rate [mean (range)] | 1.92 (0.51-6.00) | 1.83 (0.51-6.00) |
| Transmission rate ratio (badger-cattle/cattle-badger) [median (95% HPD)] | 2.1 (95% HPD: 0.8-3.8) | 1.5 (95% HPD: 0.7-2.3) |
| Median Sampled/unsampled cases (Case finding %) | 89.5/130.1 (43.2%) | 94.5/108.5 (46.6%) |

**Table A. Lengths of SNP alignments generated in this study**

| Dataset | Isolates (n) | Length of SNP alignment (bp) |
| --- | --- | --- |
| **RBCT** | 1442 | 3565 |
| **Eu1** | 4282 (including *M. caprae* outgroup) | 36600 |
| **Cluster 1** | 161 | 171 |
| **Cluster 2** | 54 | 95 |
| **Cluster 3** | 83 | 136 |
| **Cluster 4** | 86 | 91 |
| **Cluster 5** | 97 | 103 |
| **Cluster 6** | 139 | 123 |
| **Cluster 7** | 92 | 70 |
| **Cluster 8** | 110 | 174 |
| **Cluster 9** | 193 | 247 |
| **Cluster 10** | 87 | 127 |
| **Cluster 11** | 64 | 62 |
| **Cluster 12** | 58 | 49 |

**Table B. Model performance based on Maximum Likelihood Estimates (MLE) and Bayes Factors for all transmission clusters**

| **Transmission cluster** | **Model** | **log MLE** | **log Bayes Factor** | **Strength of Evidence (Kass & Raftery, 1995)** |
| --- | --- | --- | --- | --- |
| **Cluster 1** | Relaxed exponential | -5536634 | 0.056 | Not worth more than a bare mention |
|  | Relaxed constant | -5536655 | 21.289 | Very strong |
|  | Strict exponential | -5536634 | - | - |
|  | **Strict constant** | **-5536659** | **25.277** | **Very strong** |
| **Cluster 2** | Relaxed exponential | -5534141 | - | - |
|  | Relaxed constant | -5534156 | 14.906 | Very strong |
|  | Strict exponential | -5534146 | 4.800 | Strong |
|  | **Strict constant** | **-5534161** | **19.609** | **Very strong** |
| **Cluster 3** | Relaxed exponential | -5535466 | - | - |
|  | Relaxed constant | -5535498 | 31.000 | Very strong |
|  | Strict exponential | -5535468 | 2.014 | Positive |
|  | **Strict constant** | **-5535505** | **39.717** | **Very strong** |
| **Cluster 4** | Relaxed exponential | -5534382 | 0.101 | Not worth more than a bare mention |
|  | Relaxed constant | -5534394 | 12.002 | Very strong |
|  | Strict exponential | -5534382 | - | - |
|  | **Strict constant** | **-5534396** | **13.793** | **Very strong** |
| **Cluster 5** | Relaxed exponential | -5534469 | - | - |
|  | Relaxed constant | -5534474 | 4.936 | Strong |
|  | Strict exponential | -5534470 | 1.661 | Positive |
|  | **Strict constant** | **-5534476** | **7.052** | **Very strong** |
| **Cluster 6** | Relaxed exponential | -5535426 | - | - |
|  | Relaxed constant | -5535452 | 25.594 | Very strong |
|  | Strict exponential | -5535428 | 2.099 | Positive |
|  | **Strict constant** | **-5535453** | **27.358** | **Very strong** |
| **Cluster 7** | Relaxed exponential | -5533415 | - | - |
|  | Relaxed constant | -5533417 | 2.257 | Positive |
|  | Strict exponential | -5533421 | 5.893 | Very strong |
|  | **Strict constant** | **-5533421** | **6.083** | **Very strong** |
| **Cluster 8** | Relaxed exponential | -5535839 | - | - |
|  | Relaxed constant | -5535863 | 23.890 | Very strong |
|  | Strict exponential | -5535841 | 2.074 | Positive |
|  | **Strict constant** | **-5535866** | **26.912** | **Very strong** |
| **Cluster 9** | Relaxed exponential | -5538088 | - | - |
|  | Relaxed constant | -5538147 | 59.427 | Very strong |
|  | Strict exponential | -5538088 | 0.389 | Not worth more than a bare mention |
|  | **Strict constant** | **-5538157** | **69.010** | **Very strong** |
| **Cluster 10** | Relaxed exponential | -5534887 | - | - |
|  | Relaxed constant | -5534891 | 3.526 | Strong |
|  | Strict exponential | -5534888 | 0.799 | Not worth more than a bare mention |
|  | **Strict constant** | **-5534894** | **7.076** | **Very strong** |
| **Cluster 11** | Relaxed exponential | -5533358 | 1.383 | Positive |
|  | Relaxed constant | -5533358 | 0.961 | Not worth more than a bare mention |
|  | Strict exponential | -5533357 | - | - |
|  | **Strict constant** | **-5533361** | **3.610** | **Strong** |
| **Cluster 12** | Relaxed exponential | -5533082 | 1.546 | Positive |
|  | Relaxed constant | -5533081 | - | - |
|  | **Strict exponential** | **-5533085** | **4.393** | **Strong** |
|  | Strict constant | -5533083 | 2.369 | Positive |

**Table C. Effective Sample Size (ESS) for TransPhylo parameters.**

| **Transmission cluster** | **Sampling proportion pi** | **Within-host coalescent rate Ne*** | **Basic reproduction R** |
| --- | --- | --- | --- |
| **Cluster 1** | 1721 | 367 | 2407 |
| **Cluster 2** | 6027 | 1119 | 3400 |
| **Cluster 3** | 1890 | 391 | 3196 |
| **Cluster 4** | 4761 | 581 | 2649 |
| **Cluster 5** | 108783 | 933 | 10035 |
| **Cluster 6** | 2477 | 322 | 4910 |
| **Cluster 7** | 86600 | 568 | 3311 |
| **Cluster 8** | 3393 | 720 | 13012 |
| **Cluster 9** | 10640 | 202 | 17844 |
| **Cluster 10** | 2620 | 150 | 3533 |
| **Cluster 11** | 22204 | 1579 | 5094 |
| **Cluster 12** | 11576 | 8014 | 3128 |

**Table D. Substitution rates for each transmission cluster**

| **Transmission cluster** | **Median substitution rate (substitutions/site/year)** | **Median substitution rate (substitutions/genome/year)** |
| --- | --- | --- |
| **Cluster 1** | 4.3 x 10^-7^ | 1.86 |
| **Cluster 2** | 2.1 x 10^-7^ | 0.89 |
| **Cluster 3** | 1.4 x 10^-6^ | 6.00 |
| **Cluster 4** | 8.8 x 10^-7^ | 3.82 |
| **Cluster 5** | 3.5 x 10^-7^ | 1.53 |
| **Cluster 6** | 9.1 x 10^-7^ | 3.94 |
| **Cluster 7** | 1.2 x 10^-7^ | 0.52 |
| **Cluster 8** | 2.0 x 10^-7^ | 0.87 |
| **Cluster 9** | 3.2 x 10^-7^ | 1.39 |
| **Cluster 10** | 2.4 x 10^-7^ | 1.04 |
| **Cluster 11** | 1.5 x 10^-7^ | 0.66 |
| **Cluster 12** | 1.2 x 10^-7^ | 0.51 |


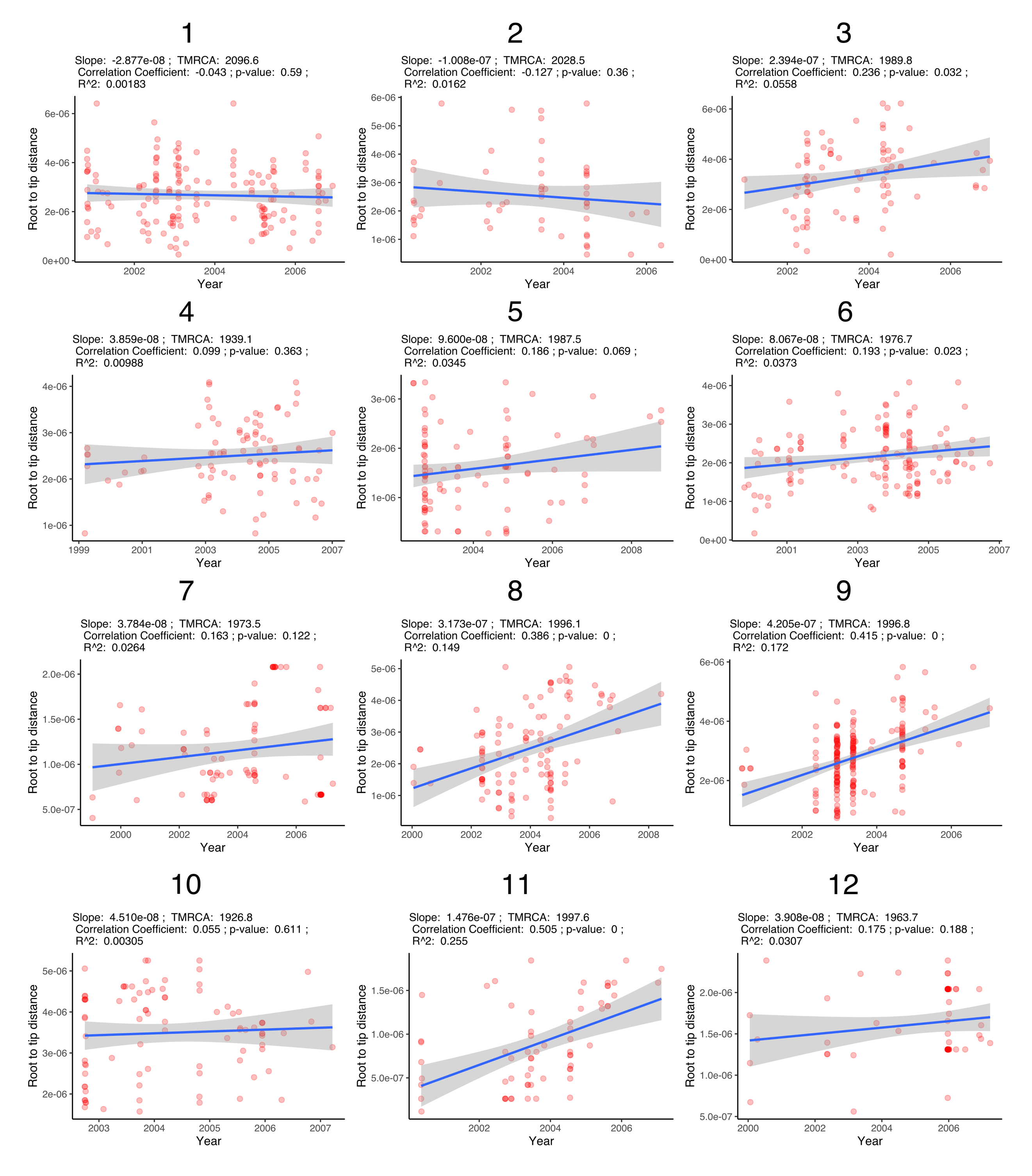


**Fig A. Root to tip distances plotted against sampling dates for all isolates in each transmission cluster.**


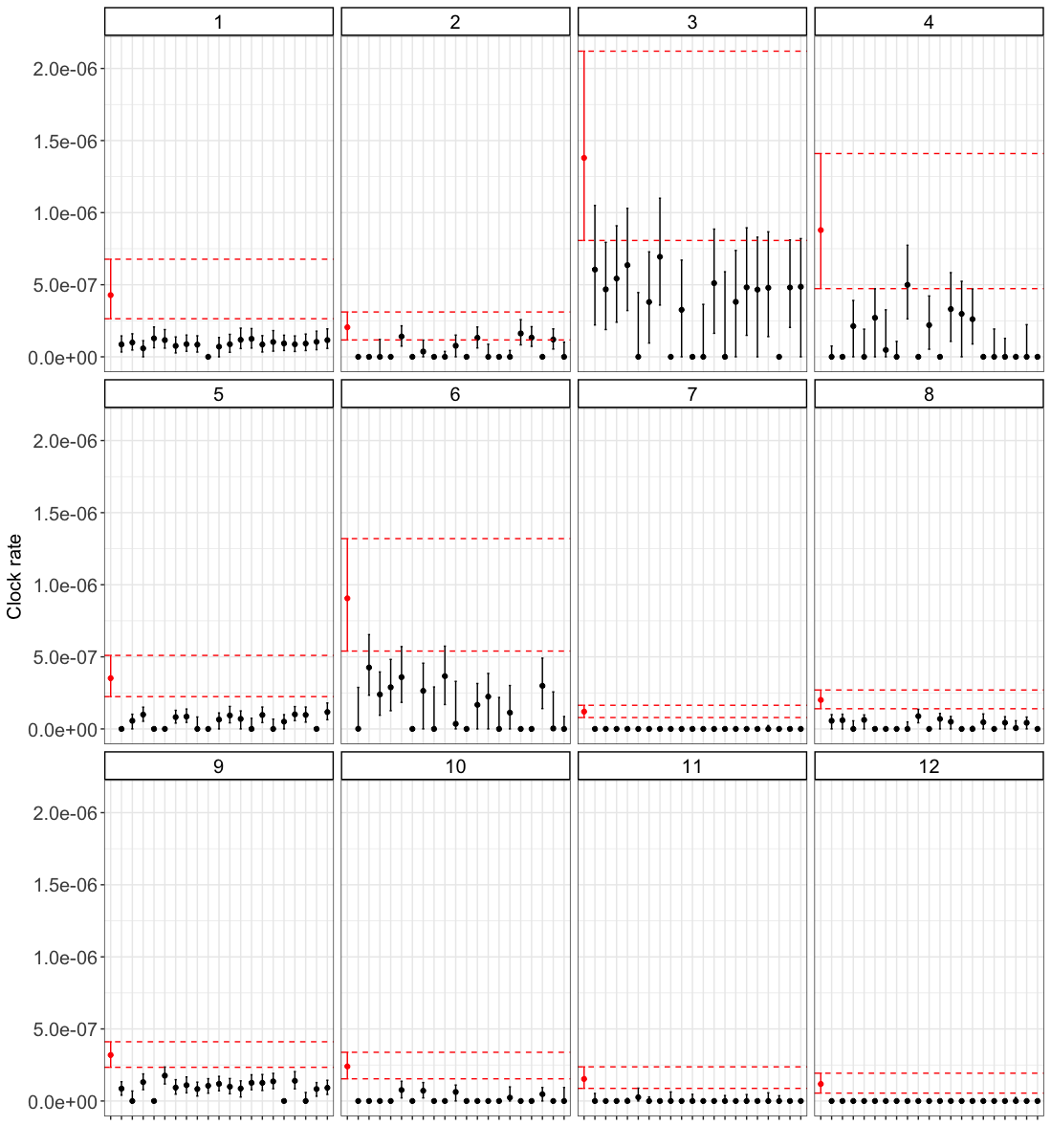


**Fig B. Date randomization (DTR) analysis in BEAST for each transmission cluster. Estimated substitution rates (mean and highest posterior density) shown in red for the observed dataset and black for the randomized datasets.**


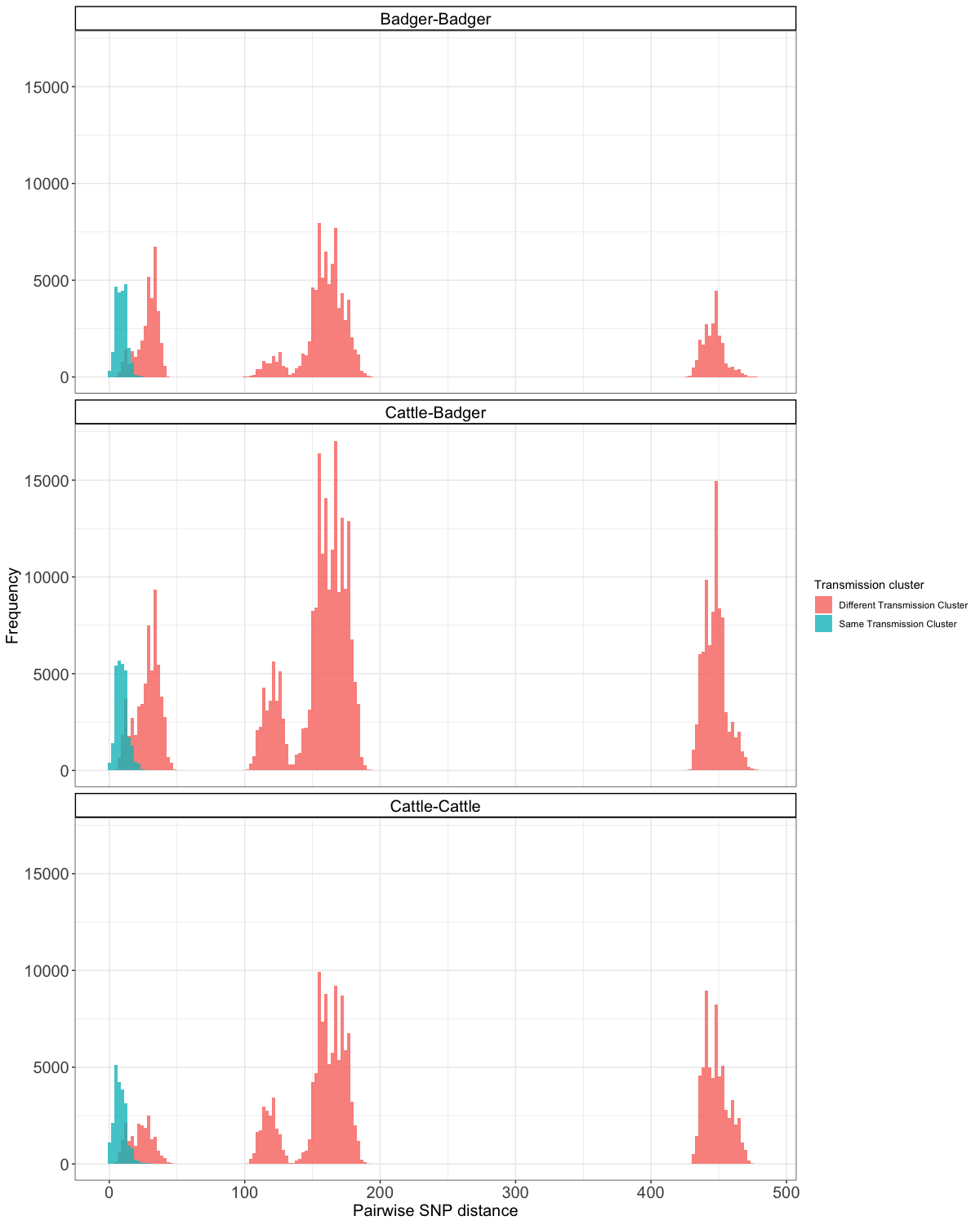


**Fig C. Pairwise distance histograms for all samples, coloured by between/within transmission cluster and separated by host pair.**


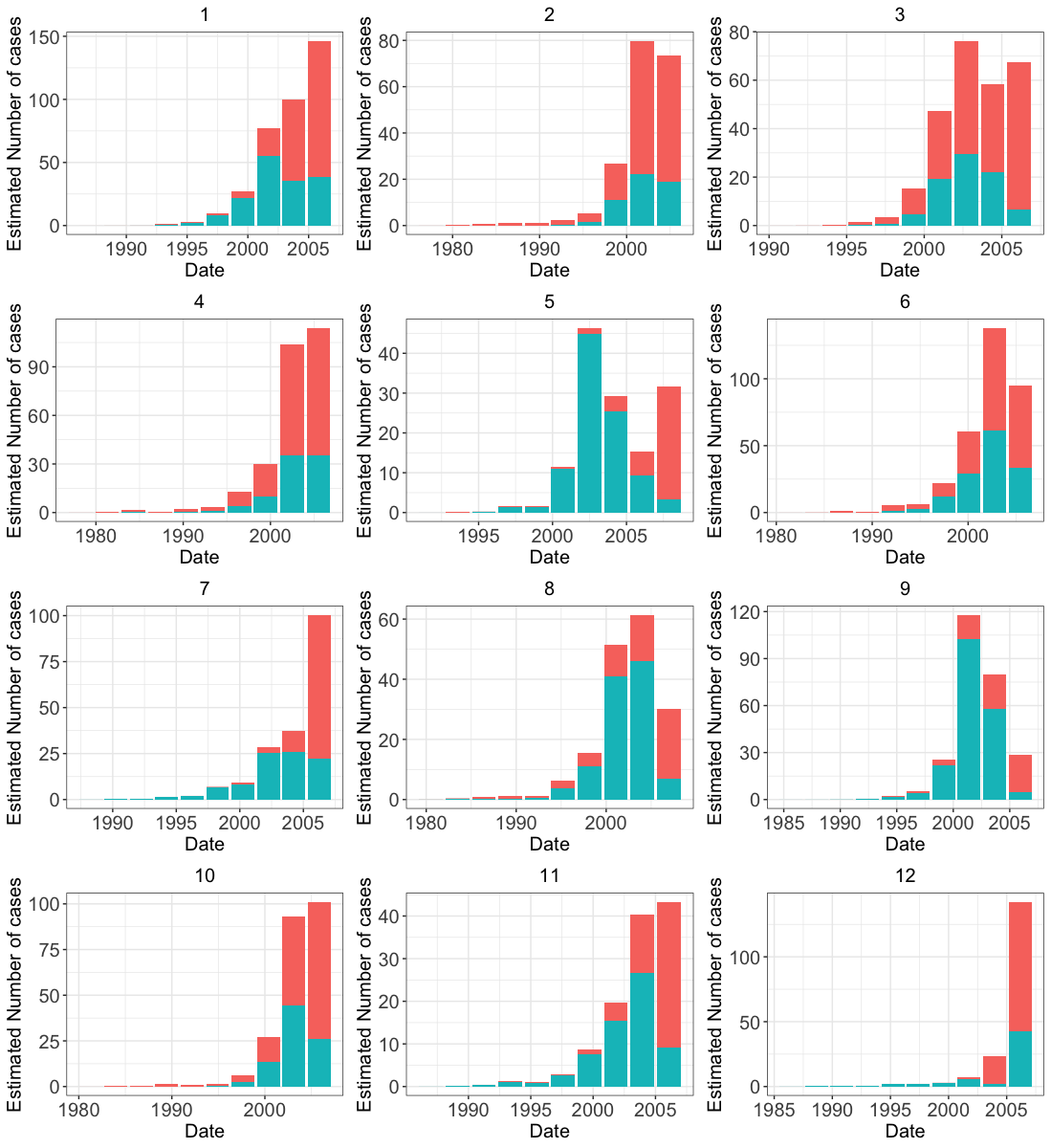


**Fig D. Proportion of sampled and estimated unsampled cases for each transmission cluster. Sampled and unsampled cases are shown in red and blue respectively**
